# Service providers’ perceptions of support needs for Indigenous cancer patients in Saskatchewan: a needs assessment

**DOI:** 10.1186/s12913-021-06821-6

**Published:** 2021-08-21

**Authors:** Jennifer R. Sedgewick, Anum Ali, Andreea Badea, Tracey Carr, Gary Groot

**Affiliations:** Department of Community Health and Epidemiology, University of Saskatchewan, Room 3242, Health Science Building 107 Wiggins Road, Saskatoon, SK S7N 5E5 Canada

**Keywords:** Indigenous, Needs assessment, Cancer supports, Service providers, Health disparities

## Abstract

**Background:**

In Saskatchewan, Canada, Indigenous cancer care services at the municipal, provincial, and federal levels are intended to improve quality care but can result in a complex, fragmented, and multi-jurisdictional health care system. A multi-phase needs assessment project was initiated to document Indigenous cancer care needs. Guided by Indigenous patient partners, clinicians, academics, and policy makers, the present study reflects a needs assessment of Indigenous cancer supports from the perspectives of cancer care service providers.

**Methods:**

Qualitative data were collected through three focus groups with 20 service providers for cancer patients and their families at three Saskatchewan cities. Participants included chemotherapy and radiation nurses, social workers, a patient navigator, dieticians, and practicum students. A semi-structured interview guide was used to conduct the sessions to allow for freedom of responses. Data were recorded, transcribed verbatim, and analyzed using thematic analysis.

**Results:**

Service providers’ perspectives were categorized into five themes: 1) addressing travel-related issues, 2) logistical challenges, 3) improvements to Indigenous-specific health care supports, 4) cultural sensitivity in health care, and 5) consistency in care. Supports provided differed for the two Indigenous groups, First Nations and Métis. Service providers made recommendations regarding how needs could be met. They saw language translation providers and Elder supports as important. Recommendations for improving travel were for medical taxis to include breaks so that passengers may alleviate any uncomfortable side effects of their cancer treatment. Further, Indigenous-specific accommodations were recommended for those requiring medical travel. These recommendations aligned with supports that are available in four other Canadian provinces.

**Conclusions:**

These results identified gaps in supports and outlined recommendations to address barriers to cancer care from the perspectives of service providers. These recommendations may inform evidence-based health system interventions for Indigenous cancer patients and ultimately aim to improve cancer care services, quality of life, and health outcomes of Indigenous patients throughout their cancer journey.

**Supplementary Information:**

The online version contains supplementary material available at 10.1186/s12913-021-06821-6.

## Background

First Nations, Inuit, and Métis are ethnic groups that comprise Indigenous peoples in Canada. Within these groups, the rates of cancer diagnoses are disproportionately rising [1, 2] and their survival rates are comparatively lower than Canada’s non-Indigenous population [3]. In the province of Ontario, the rate of new cancer cases from 1968 to 2001 for First Nations people has nearly doubled [4]. From Ontario’s health status data from 2001 to 2010, new cancer diagnoses and mortality rates continue to be significantly higher for First Nations in comparison to non-First Nations Ontarians [5]. These outcomes extend beyond Ontario, as data from 2004 to 2011 in the province of Manitoba reveal that First Nations (vs non-Indigenous Manitobans) were diagnosed with cancer significantly younger, had higher late-stage cancer diagnoses, and had higher mortality rates [6]. Further, First Nations in Manitoba living on reserve had higher proportions of late-stage diagnoses than First Nations living off reserve [7]. These findings suggest that cancer is an increasingly relevant health issue for Indigenous people in Canada, making optimal care for Indigenous cancer patients a significant concern to address.

Cancer diagnosis, treatment, and aftercare may be especially challenging for Indigenous patients and their families. Regarding health coverage, First Nations people registered under the *Indian Act* and Inuit recognized by an Inuit land claim organization are covered through the Non-Insured Health Benefits (NIHB) program that is provided by the federal government’s Department of Indigenous Services Canada [8]. Specific to cancer care, coverage includes medical supplies and equipment, medical transportation, and reimbursement for accommodations in circumstances when health services are not locally available. However, Métis are not eligible for this coverage because they are not under the federal jurisdiction of the *Indian Act.* Instead, Métis registered through one of the five provincial Métis organizations can apply for coverage for relevant initiatives if their organization offers such supports, and otherwise receive the same coverage as non-Indigenous Canadians. Aside from medical coverage, municipal health care centres and provincial health organizations may offer additional Indigenous-specific programs to provide services such as cultural and spiritual support (e.g., Elder support, translation services). The presence of Indigenous health programs and services at the municipal, provincial, and federal levels are intended to ensure improved quality care but can result in a health care system that is complex, fragmented, and multi-jurisdictional [9]. Consequently, cancer diagnosis, treatment, and aftercare may be especially challenging for Indigenous patients and their families.

Geographical differences are an additional challenge for many Indigenous cancer patients. According to the 2016 Canadian census, 38.9% of Indigenous people live in rural areas, 20% in small population centres (1000–29,999 people), 10.8% in medium centres (30,000–99,999 people), and 30.3% in large population centres (over 100,000 people) [10]. Therefore, the majority of Indigenous peoples must travel to receive treatment, as cancer treatment centres are predominantly in cities with large population centres. To address geographical barriers, the NIHB program and some Métis Nation organizations offer coverage and support for Indigenous patients required to travel [8, 11]. However, despite general supports and some Indigenous-specific cancer services, there is evidence of unmet needs for cancer support services for the Indigenous population [9, 12–14].

Prior to our research, there had been no systematic assessment of the cancer care support services for Indigenous people in the province of Saskatchewan. To address this gap, a multi-phase needs assessment project was initiated titled S*âkipakâwin*, the Cree^1^ word for budding or sprouting. This research project was guided by an advisory team of Indigenous patient partners, clinicians, academics, and policy makers, whereby the phases were to conduct a scan of the current Indigenous-specific cancer supports in Canada, a sharing circle study with Indigenous people affected by cancer, and a study with service providers to examine perspectives on barriers and supports for Indigenous cancer patients. Separate studies were selected for Indigenous cancer patients and service provider participants to address potential power imbalances between patients and providers and because of the importance of using a culturally-relevant data collection method with Indigenous participants (e.g., sharing circles) [12, 15].

The present study represents the latter aim of S*âkipakâwin* by using a needs assessment methodology [16] to identify the support needs of Indigenous cancer patients and their families from the perspectives of cancer service providers. Identifying and prioritizing these needs are the first steps to implementing evidence-based health system interventions in supporting Indigenous patients and their families during cancer diagnosis, treatment, and follow-up.

## Methods

### Setting

Data were collected from the three sites with the most extensive cancer supports and numerous cancer service providers in the province: the Saskatoon Cancer Centre, the Allan Blair Cancer Centre in Regina, and the Community Oncology Program of Saskatchewan (COPS) in Prince Albert. These centres are operated by the Saskatchewan Cancer Agency (SCA) and are separate from the hospitals that are run by the provincial health organization, the Saskatchewan Health Authority (SHA). Services that they provide include facilitating prevention and early detection programs, providing cancer screening, and delivering non-surgical cancer treatments (e.g., chemotherapy, radiation therapy) [17].

With regards to the Canadian province selected as the setting of the study, Saskatchewan is an exemplary choice because it is the province with the second highest proportion of Indigenous peoples (16.3%) and the majority of this population live in remote, rural, or small population centres (62%) [10]. Of the Indigenous population, 65.5% are First Nations (65.5% Registered Indian; 34.5% non-Registered Indian) and 33.1% are Métis (remaining proportion represent those with multiple Indigenous identities). Representation of these specific groups are important, as each group receives different medical coverage and consequently, may experience distinct barriers to accessing care.

### Participants

For the current study, qualitative data were collected from 20 cancer service providers using a focus group approach. Providers included chemotherapy and radiation nurses, social workers, a cancer navigator, a dietician, and practicum students, and providers’ length of time working in cancer care ranged from 6 months to 20 years (see Table 1 for participant characteristics). Criterion sampling was used whereby the target participants, health care professionals from cancer care facilities (e.g., cancer centres, COPS centres), were invited to participate by email through the research coordinator and managers from cancer units employed by the SCA. To note, both Indigenous and non-Indigenous providers were invited to participate. Service providers could choose whether they wanted to participate by attending the scheduled study session. All sessions took place over the lunch hour to maximize the number of service providers that would simultaneously be available to participate.

**Table 1:** Participant Characteristics

Informants: ***n*** = 20	
**Cancer Centre Site**	
*Allan Blair Cancer Centre*	8
*Saskatoon Cancer Centre*	6
*Prince Albert COPS centre*	6
**Service Provider Type**	
*Chemotherapy and radiation nurses*	9
*Social workers*	4
*Practicum students*	5
*Cancer navigator*	1
*Dieticians*	1
**Gender**	
*Women*	18
*Men*	2
**Indigenous Status of Participants**	
*Non-Indigenous*	19
*Indigenous*	1

Aside from a meal that was provided during the focus groups, no compensation was offered for participating in the study. All participants eligible to participate attended the study session except for two in Regina, three in Saskatoon, and one in Prince Albert. Research ethics approval was provided by the Behavioral Research Ethics Board (REB) at the University of Saskatchewan (#18–105).

### Procedure

Focus groups were facilitated by two researchers in the aforementioned cancer care settings. After participants provided informed consent, data collection began by asking general questions about Indigenous people within the health care system (e.g., “How do you identify that a patient is Indigenous?”). A semi-structured interview guide was used to conduct the remainder of the session to allow for freedom of responses. Questions were specific to participants’ perspectives on the support needs of Indigenous cancer patients and their ability to provide the needs relevant to their profession (see Additional file 1: Appendix for full list of interview questions).

Consistent with the focus group method [18, 19], questions were either posed by the researchers or participants organically responded to the questions through ongoing group discussion. Probing questions were asked when necessary to ensure that each question was answered and that responses were complete. Data were collected using an audio recorder and interviews were transcribed verbatim by the University of Saskatchewan’s Social Sciences Research Lab. Each focus group was approximately one hour.

After data from the first two focus groups were collected, transcribed, and coded, we identified consistent themes across the study sites and recognized that we had reached data saturation [20]. However, the third focus group at an additional site was included to obtain an adequate sample size. The final themes were distributed via a technical report to the advisory team and other stakeholders including senior leaders in Indigenous governments in Saskatchewan, the SCA, and the SHA [21].

### Analysis

Transcripts were reviewed by the researchers and analyzed in ATLAS.ti 8 using a thematic analysis approach. The transcript from each focus group was independently coded by two researchers (authors JS and AA) and final themes were reviewed by the coders and author TC. Coding was conducted and validated using Braun and Clarke’s (2006) approach [22]: initial codes were identified and collated into potential themes, themes were reviewed and potential coding conflicts were addressed, and analysis was finalized by generating descriptive names for themes and subthemes. Braun and Clarke’s 15-point checklist of criteria for good thematic analysis was also used [22]. Criteria includes ensuring that researchers were active in the research process, that balance between analytical narratives and illustrative extracts are provided, and that data were interpreted rather than simply described.

## Results

**Fig. 1:**
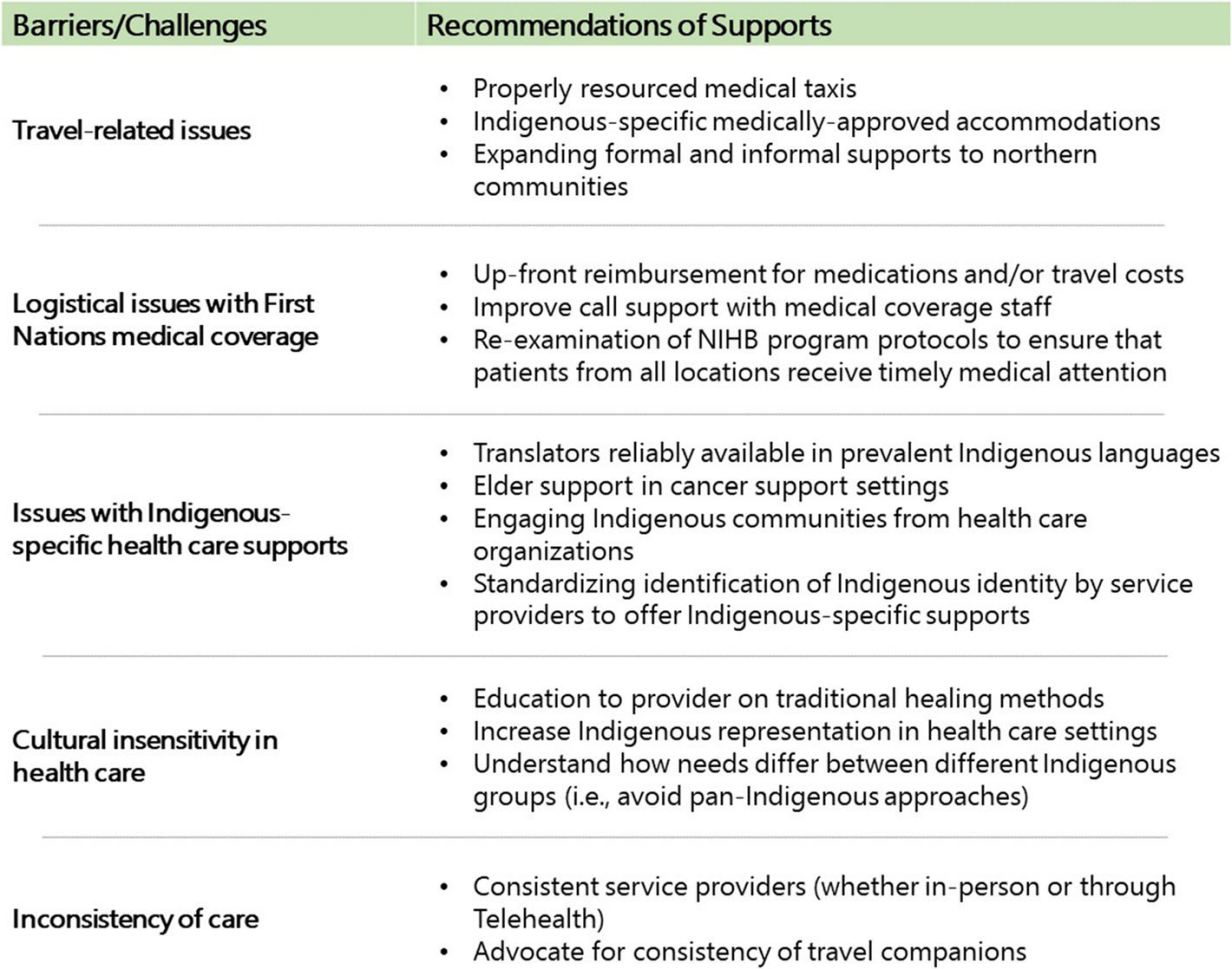
Overview of barriers and recommendations from service provider participants of Indigenous cancer support needs

Service providers shared their perceptions of barriers to cancer care for Indigenous people in Saskatchewan and discussed support needs that correspond to the barriers. Needs were identified and categorized into five themes: 1) addressing travel-related issues, 2) addressing logistical challenges with Indigenous-specific medical coverage, 3) improvements to Indigenous-specific health care supports, 4) cultural sensitivity in health care, and 5) consistency in care. To note, participant quotes in the following section will reference the focus group (FG) and participant (P) numbers. For a synthesized overview of the connection between the barriers and recommendations of Indigenous cancer support needs, see Fig. 1.

### Theme one: addressing travel-related issues

Challenges regarding transportation and accommodations were identified for Indigenous patients living in rural and remote communities. Support needs in light of these challenges are summarized in the following sub-themes: properly resourced medical taxis, Indigenous-specific accommodations, and expansion of formal and informal supports to northern communities.

#### Properly resourced medical taxis

Participants noted from previous discussions with First Nations cancer patients that there were concerns with the medical transportation service provided by the NIHB. Concerns ranged from issues relevant to all patients, such as passengers smoking inside the medical taxi, to issues specific to passengers with cancer, such as a lack of properly resourced vehicles or travel itineraries that did not meet the unique needs of cancer patients. For example, patients undergoing chemotherapy may require more rest stops in order to alleviate the side effects of their treatment, and patients with cancers that may be particularly uncomfortable during travel (e.g., anal cancer) should also be accommodated (e.g., more rest stops, appropriate seating). The following excerpt relays the experience of using medical taxis as recounted by one participant:

*I’ve had people tell me that they’re crammed into a vehicle of some kind, and if you’ve got some side effects like diarrhea and that kind of thing along the way there’s no place to stop and go to the bathroom. They’re not feeling well and they’re on some crappy road that is [a] very long distance…* (FG 1, P 4).

Participants suggested that NIHB medical transportation ought to provide support beyond its utility of transport by inquiring about and accommodating the specific travel needs of cancer patients.

#### Indigenous-specific accommodations

Accommodations for cancer patients known as “cancer lodges” were recognized to be underutilized supports by Indigenous patients despite being the only accommodations that cater to the needs of cancer patients undergoing treatment. Several participants relayed that First Nations and Métis patients felt uncomfortable and speculated that this could be from experiencing racism within these lodges (“… *I mean, we don’t have control over the other people who stay there, and maybe they’re racist. I’ve heard that from several patients, Indigenous ones, that that was an uncomfortable place for them to stay…”*; FG 1, P 5). Rather than staying at the cancer lodges, patients were instead finding refuge elsewhere (e.g., hotels). Participants from the COPS centre in Prince Albert noted that their city was unique in that it has an Indigenous-specific boarding home, Spruce Lodge, that offers medically-approved lodging and transportation to registered First Nations patients. Whether patients chose to stay at hotels or in the boarding home, however, participants indicated that these alternative accomodations also have their limitations:

*The problem with Spruce Lodge for our cancer patients is that it is a shared bathroom, so you’re exposing everybody in Spruce Lodge to chemo, and you’re also exposing those patients to everybody else’s bacteria and stuff like that… Now it has shifted that a lot of the chemo patients will stay in hotels as opposed to staying at Spruce Lodge, which has worked out a lot better. However, depending on which hotel they stay at is dependent on how much sleep they get the night before kind of thing. Cause lots of our people will come in the day before, and we’ve had people say, “I just didn’t sleep last night, we stayed at whatever hotel and it was loud in the hotel”.* (FG 2, P 4).

Providers suggested that accommodation issues could be improved through Indigenous-specific lodging that accommodated patients undergoing cancer treatments (“*If we can’t provide [cancer care] in the community, then the least we can do is accommodate here some place*”; FG 1, P 2).

#### Expansion of formal and informal supports to northern communities

Service providers across all focus groups highlighted detrimental consequences often experienced by patients who must travel long distances for extensive periods of time to receive cancer treatment. For instance, being away from the community holds psychosocial implications for Indigenous patients that can compromise their willingness to receive treatment. This circumstance is illustrated with the following quote:

*…I did work for a northern health organization… where we would have people who would just choose not to get treatment because of the time that it would take them away from their community. Just the difficulties with family and the community, you know, being away from home.* (FG 3, P 5).

Considering the lack of community support and the other barriers with medical travel previously discussed, providers determined that formal supports, such as some cancer treatments, and informal supports, such as home care and nutrition services, requires northern expansion (e.g., “*So all of our treatment facilities are basically south of halfway of our province. So yeah, additional COPS centres [community oncology programs] or health facilities, in more locations*” [FG 1, P 2]). Other proposed solutions to reducing medical travel included ensuring that the province’s medical appointment platform, TeleHealth, was functional (“*It’s not working consistently, it’s not a reliable tool to be used”*; FG 2, P 4) so that it could be effectively utilized, and to train health care providers in northern communities to deliver similar services. One patient notes:

*Some of the communities aren’t comfortable, I guess, with removing those lines and flushing those lines appropriately. As a result, those patients have to remain with us for a couple days so that homecare here in the city can remove the line… So education to the communities of how to care for these lines would be valuable I think.* (FG 3, P 3).

Together, participants determined that further training for northern health care providers on out-patient cancer care, reliable medical appointment technology, and expansion of cancer treatment programs in the north would reduce the need for patients to travel from their communities for cancer care.

Theme two: Addressing logistical challenges with Indigenous-specific medical coverage.

A significant focus of each focus group concerned the complexities of Indigenous patients accessing existing supports, and that these complexities often compromised the benefits that the supports were intended to deliver. Two sub-themes emerged regarding needs that addressed logistical issues: coordinating care with NIHB (travel, prescriptions) for First Nations patients and reimbursing First Nations patients for medications and/or travel up-front.

#### Coordinating care with NIHB (travel, prescriptions) for First Nations patients

Although medical coverage through the federal government’s NIHB program is intended to provide support (e.g., travel coverage) for registered First Nations and Inuit, participants expressed their frustrations when having to contact employees of the program to advocate for unmet patient supports. For instance, necessary supports were often denied or were restricted by the program’s rigid regulations. One provider explained that they received different responses for identical inquiries from NIHB program employees when advocating for patients that were denied medical travel from their accommodations:

*Well on one phone call I may have to advocate for taxis in town, whereas the next one I’m getting told “no, because they’re close enough to the hospital that you shouldn’t need a taxi.” So then I’m fighting about that… So if we don’t know the system, then our patients aren’t getting the services needed.* (FG 1, P 1).

Further complicating providers’ advocacy efforts is the extensive time often required to communicate with those who run the program when coordinating coverage:

*…my longest [time on hold on the phone] was an hour and 21 min waiting to get through to them. So that’s formal resources being used inappropriately. When you finally get through to them, sometimes they can’t help – because you’re phoning a call center, these aren’t people that dedicated their lives to Indigenous issues.* (FG 1, P 1).

A second provider note that an additional weakness of the NIHB program’s communication model is that there is no consistent contact with employees. They state that this leads to time loss because providers are “*repeatedly calling [the NIHB Program] back*” regarding the same patient and that they would often be “*getting a different person”* and therefore *“taking a lot of time to re-explain the situation and re-advocating for the same things*”. (FG 1, P 2).

An additional travel-related issue regards the relatively limited timeframe in which travel coverage can be coordinated, as medical travel provided through the NIHB must be arranged at least 2 weeks prior to the appointment. Participants mention that the timeframe required to schedule medical travel was particularly burdensome if care was urgent or if a last-minute appointment time became available to First Nations patients. The limited timeframe to submit requests for NIHB-covered patients also extended to receiving medications, indicating that this logistical issue is impeding participants from accessing their medical needs.

#### Reimbursing First Nations patients for medications and/or travel up-front

Participants noted an economic barrier experienced by some registered First Nations patients was the delayed medical coverage for their cancer-related expenses. A discussion between participants illustrates this issue (FG 2):

P1: …*there was lots of First Nations who travelled from reserves that were 800 km from Saskatoon so then they need to fly in, they need to have a hotel and stay overnight – it’s a major expense for an individual. It seemed to be a somewhat complicated funding structure because they’d go through the band office and that would have to be approved. It never seemed easy for those people to get to the clinic.*

*P2: Sometimes it’s a fight to have that money come up front and then have to submit all that paper or receipts or whatever and then get that payback. But that’s subjective to each nation, right?*

*P3: And if they don’t have the actual money to put up front-.*

*P1: Yeah, some don’t even have it.*

*P3: They can’t go, ‘cause getting money after they come back isn’t doing them any good, ‘cause they haven’t any to get here.*

As stated in the dialogue, travel or medical coverage through NIHB may still not be accessible to First Nations patients from low-income households because of the up-front costs. Participants agreed that the current model of medical coverage is not adequate to accommodate the patients who require this support the most. Instead, expenses should be covered directly through the funding body (e.g., the NIHB Program).

### Theme three: improvements to Indigenous-specific health care supports

Participants identified limitations within existing services available to Indigenous patients within hospitals and other health care facilities in the province. The following sub-themes outline proposed improvements to: Indigenous language interpreters, Elder support, and standardizing identification of Indigenous identity to ensure that Indigenous-specific health care supports can be offered.

#### Indigenous language interpreters

Adequate interpretation was repeatedly discussed, with one participant asserting that “*language is one of our biggest barriers, and trying to rectify that would be a huge step in the right direction*”. (FG 1, P1) Providers acknowledged that interpretation was offered for some First Nations languages but noted limitations with translation services. Despite having access, there were not enough translators to meet the demand of patients. Recommendations were to increase the number of interpreters and include more Indigenous languages for language translation. One participant said: “*Yeah, we do have regular struggles with Dene*^2^*because it’s – We don’t have as many. Like we have a few, even staff that can speak Cree in the building at any given time. But Dene seems to be the more common struggle*”. (FG 3, P 5).

An additional concern was that translators were not immediately offered to Indigenous patients with medical companions. Participants expressed that translators were necessary because of their training in providing medical translation, which is a skill that medical companions do not necessarily have. The following participant states their recommendation:

*I almost wonder too, yeah, if just – especially maybe their first appointment with an oncologist in Saskatoon, if there’s a translator that’s not family present at that, and it’s just not questioned. This is just how we do things, this is how it is, they’re a medically trained translator, they’re here no matter what, kind of thing.* (FG 3, P 4).

A final proposed solution to address issues with access to language translation supports models a current service offered to patients who speak non-Indigenous languages. The provincial health agency uses MCIS Language Services, an external organization that provides professional interpretation services 24 h a day, 7 days a week for patients. This organization does not offer translation in any Indigenous languages, but a comparable system was proposed. The following details this service by one participant:

*It has to be pre-arranged. There’s a 1–800 number. I mean you could contact them and you may get same day service, it’s just not immediately... Because even with Cree and Dene, a lot of things are difficult, it’s not just the language it’s the medical aspect… then not only are they [the telephone interpreter] fluent in the language, but also fluent in medical language too.* (FG 3, P 5).

#### Elder support

Elder support offered to Indigenous patients through the province’s First Nations and Métis Health Services was praised by participants for providing patients with advocacy, representation in health care, and culturally relevant supports. One participant notes:

*He’s amazing and he’s got lots of knowledge, he helps guide - he’s one of those people who’s a key - there’s the traditional pathways for Indigenous pathways and then we have the mainstream way of doing things, he serves as a “Let’s find a way for both*”. (FG 2, P 5).

However, Elder support is exclusively offered in hospitals because the cancer centres are separate institutions, and those using medical travel through the NIHB program are only provided transportation to and from the location where the patient received cancer care. Consequently, First Nations patients using medical travel may not have access to Elder support.

Providers also discussed needs for stronger relations between the provincial health agency and influential members within Indigenous communities, such as Elders. The advantage is that Elders can assist with advocating for health and wellness through education such as cancer prevention. To facilitate this, health officials need to “*try to bridge the gap of getting some buy-in from the community level*” in order to have community members advocate *“to try and help get people out and motivate them to take action for their health…”.* (FG 2, P 4).

#### Standardizing identification of Indigenous identity to ensure that Indigenous-specific health care supports can be offered

Although service providers can inform Indigenous patients of Indigenous-specific supports from First Nations and Métis Health Services, participants from all focus groups stated that no systematic approach existed for identifying Indigenous patients who would otherwise benefit from these supports. The lack of formal approach was evident from the varied answers that providers gave when asked how they determine if a patient is Indigenous. Answers included patient’s self-identification, asking the patient, judgements about the patient (e.g., racial identity, last name, home address), and from observing registration numbers of Indian status cards from First Nations patients’ electronic charts. The lack of reliable approach is problematic because individuals can be Indigenous without physical characteristics and last names provide no guarantee of Indigeneity. Further, a significant proportion live in large population centres (27.5% in Saskatchewan) [8], and other Indigenous groups such as Métis do not have Indian Status and thus do not have registration numbers. Participants concluded that the presence of Indigenous-specific services was a positive support, but that an emphasis on a methodical approach to identifying Indigenous patients was needed.

### Theme four: cultural sensitivity in health care

Participants acknowledged that most health care providers are non-Indigenous, and that non-Indigenous providers are less likely to be informed on how to provide culturally relevant care to Indigenous cancer patients. Participants discussed how culturally relevant care could be better facilitated by the following themes: cross-cultural understanding for non-Indigenous service providers and Indigenous service providers in all stages of cancer care continuum.

#### Cross-cultural understanding for non-Indigenous service providers

Health care professionals in Saskatchewan are often obligated to complete Indigenous sensitivity training during post-secondary education or workplace in-services to facilitate cross-cultural understanding. However, as participant 5 (FG 2) states, “*It needs to be more than just an Indigenous sensitivity training that usually is typical of checked box… They facilitate a conversation about it but don’t continue… doing more initiatives”*. One initiative discussed was education on traditional healing methods to inform providers what it is, how it may be integrated with Western medicine, and how to discuss its integration with patients *(“…having a better understanding and respect for the holistic side of things too, to integrate them better, present all the options, make informed decisions”*; FG 2, P 4). Further, it could address stigma that providers may have about non-Western treatment options *(“if you know a disease is curable but they’re going to go traditional medicine, as medical professionals I think we have difficulty with that”*; FG 2, P 3).

#### Indigenous service providers in all stages of cancer care continuum

To promote cultural sensitivity in health care, discussions from all focus groups highlighted the need for Indigenous representation from cancer care providers. One participant emphasized the need for representation from both dominant Indigenous groups in Saskatchewan: *“[First Nations and Métis] have very different realities in terms of practically everything. Very different experiences, very different supports that they have access to”* (FG 2, P 7). The quote illustrates that the experiences of navigating the health care system will differ between First Nations and Métis due to the supports available to each group, but also, that their experiences differ because they are distinct cultural groups. This highlights the importance of avoiding a pan-Indigenous approach by ensuring that needs (e.g., representation) are met for the predominant Indigenous groups in the province.

### Theme five: consistency in care

Participants issued concerns relating to inconsistencies in both formal and informal care providers. The consequences of inconsistencies are among the following two themes: consistency from service providers (i.e., formal supports) and consistency from travel companions (i.e., informal supports).

#### Consistency from service providers (i.e., formal supports)

Participants acknowledged that there are higher inconsistencies in care for Indigenous patients living in the rural and remote areas of the province because of unreliable virtual appointments through Telehealth. Oncologist care was noted to be particularly unpredictable for Telehealth appointments:

*They’re doctors, they’re oncologists, but they’re not their primary oncologist. And so they might go to Telehealth in January and see their primary oncologist who’ve they’ve always seen, and then they go in February, March, April, May, and they see somebody different. And it may be a different person each time, let alone just one person.* (FG 3, P 4).

Participants highlighted two virtues of consistent cancer care providers. One provider captured both strengths of consistency in the following quote:

*… I think that transition [of primary care providers] for a lot of people, especially people from the north when you talk about trust issues, that is not good to have fill-in doctors come in. They don’t have the same rapport. The stuff they talked about at one Telehealth may not ever come up again.* (FG 3, P 4).

When patients must consistently retell their experiences, they may fail to share all details and consequently, as discussed among participants, the provider would be unaware of key details that could assist the patient.

#### Consistency from travel companions (i.e., informal supports)

Travel companions are a valuable informal support to Indigenous patients and can also be helpful for conducting language translation. Despite their benefits, participants mentioned that communication issues can arise when patients bring a different medical companion for each appointment. For instance, one participant posits that patients may selectively disclose information based on which companion accompanied them:

*… I think the other problem that we’ve had too, is sometimes it is a daughter or son that comes down with their parent, or even a husband and wife, and the patient may not be comfortable talking about certain things in front of who’ve they’ve brought… Dad may be comfortable in front of one child and not in front of the other child* (FG 3, P 4).

Another provider speculates that the inconsistency of health companions may be due to the limits of the NIHB Program. They state: *“…one of the biggest things that we always say to people coming to the Cancer Clinic is, “you can bring several family members with you. Whoever’s gonna make you feel comfortable.” Often many of the First Nations people are limited to one person to be able to come. To me that’s not quite right…”.* (FG 1, P 4) To elaborate, because First Nations patients are only funded for one medical companion, the patient may be less likely to articulate their experiences to the provider in front of the sole companion. If multiple travel companions were funded to accompany the patient, family members would be less likely to rotate as travel companions and thus, facilitate better companion consistency.

## Discussion

The experience of accessing and receiving cancer care for Indigenous peoples in Canada is often distinct from the experience of non-Indigenous Canadians because of geographic barriers, navigating different and often complex systems of medical coverage, and cultural differences with the Western medical system. To accommodate for these potential differences, some provinces in Canada offer Indigenous-specific cancer supports [23], though in Saskatchewan, there were limited supports for Indigenous patients in general and no supports specific to cancer. In light of these limited supports, we conducted a needs assessment of Indigenous cancer supports through focus groups with cancer service providers. In our study, participants were aware of many unmet needs that Indigenous cancer patients experience and outlined recommendations for addressing these needs through five themes of support needs: 1) Addressing travel-related issues, 2) Addressing logistical challenges with Indigenous-specific medical coverage, 3) Improvements to Indigenous-specific health care supports, 4) Cultural sensitivity in health care, and 5) Consistency in care.

While previous studies conducted in Canada have examined First Nations’ barriers to cervical cancer screening in Ontario [24] and palliative care [25], service providers in the current study discussed their perceptions of barriers and the support needs for First Nations and Métis cancer patients. Further, this study revealed perceptions of needs related to all phases of the cancer care continuum. Parallel themes between these studies include geographic and transportation barriers [24] and culturally appropriate care [25]. Aside from these common barriers, the current study highlights additional barriers (e.g., lack of Indigenous representation within health care system) and ultimately, details the solutions to address these barriers. The current study also builds on previous research by including the cancer support needs of Métis. Considering the needs of Métis is significant because their medical coverage is akin to non-Indigenous people (i.e., provincial government coverage only) whereas First Nations’ coverage is through the federal government.

### Recommendations for policy and practice

Recommendations included improvements to existing supports for registered First Nations patients (e.g., coordinating and utilizing medical travel) and First Nations and Métis health programs. Specifically, communication with the federal government’s NIHB Program often led to lost time because of extensive wait times for service providers advocating for patients’ unmet needs. An additional recommendation for the NIHB Program was to conduct direct billing when possible for covered expenses, as paying for reimbursable cancer care needs upfront are a barrier for First Nations people with low-income. This is particularly relevant considering that poverty rates are disproportionately higher amongst Indigenous peoples in comparison to the non-Indigenous population in Saskatchewan [26]. Another recommendation was having additional resources for Indigenous supports in hospitals and cancer centres such as language translation and Elder supports. In summary, Indigenous health care supports are recommended to be inclusive of Indigenous groups and accessible to Indigenous patients that receive care outside of the facilities where these supports are located.

Considering that two-thirds of the Indigenous population in the province of study reside away from the cities in which cancer care is primarily received [10], it is unsurprising that many of the barriers discussed by participants related to geographical issues. Recommendations for travel improvements included offering medical taxis that accommodated the unique needs of cancer patients, such as extending the duration of travel to allow for more breaks to alleviate the uncomfortable side effects of cancer treatment. Accommodations for Indigenous patients were also discussed, with participants emphasizing that Indigenous patients conveyed discomfort when staying at lodgings that catered specifically to cancer patients and were instead staying in places like hotels that did not provide these specialized supports. Participants recommended that Indigenous-specific accommodations be provided. This is not an unrealistic recommendation, as four other provinces in Canada have lodging specifically for Indigenous people who must travel to receive health care (e.g., Lu’ma Native Housing Society in British Columbia; Wequedong Lodge of Thunder Bay in Ontario) [23].

Participants also recommended more Indigenous representation among service providers for offering culturally relevant support and for facilitating trust between patients and providers. Improving trust between Indigenous patients and health care providers is significant, as previous research demonstrates that Indigenous peoples tend to have mistrust towards the Western medical system [13] due to historical [27, 28] and ongoing racism experienced within the system [29]. Research with service providers in the province of Ontario demonstrates that systemic discrimination towards Indigenous patients is a barrier to health care, and also recommends the need for cultural safety training [30]. Importantly, representation should be increased for both First Nations and Métis because they are distinct cultural groups. To note, this recommendation requires initiatives from multiple health authorities or organizations because cancer care supports are often provided through different organizations (e.g., health authorities, cancer agencies).

An important context for our findings is that some issues for Indigenous patients that service providers noted would also be experienced by non-Indigenous patients; for instance, discomfort from staying in hotels (vs. cancer lodges) that are unequipped for patients with side effects of chemotherapy. The key difference is that the decisions of Indigenous patients accessing supports (e.g., accommodations) may be motivated by avoiding racism from non-Indigenous providers of formal supports. That is, using the accommodations example, the discomforts of staying in a hotel may be the same for both Indigenous and non-Indigenous cancer patients, but that Indigenous patients might choose the discomforts of a hotel over the discomforts of racism by staff or residents of the cancer lodges. Therefore, the lack of cultural safety within the health care system could amplify challenges beyond coordinating medical travel or discussing culturally relevant treatment options with service providers. This reiterates the need for interventions that address cultural safety such as Indigenous representation within the health care system and Indigenous sensitivity training for service providers.

### Limitations and future research

A limitation of our study is that the support needs were identified by predominantly non-Indigenous service providers and therefore, these needs may not be consistent with the needs proposed by those with lived experience (i.e., Indigenous cancer patients and their families). However, the barriers to care proposed in the current study address are reiterated from previous qualitative research examining the experiences of Indigenous cancer patients in Saskatchewan; for instance, issues with coordinating and up-front costs for medical travel [12], the lack of culturally appropriate accommodations [31], and the absence of information and access to Traditional medicine [32]. The shared perceptions of barriers to cancer care from both Indigenous peoples and service providers highlight that these concerns are relevant to the population utilizing the health care system and those providing care within it. A strength of our study is that it discusses barriers specific to cancer service provider perceptions, such as logistical challenges for providers coordinating coverage with the NIHB program.

Another limitation was that participants were asked to discuss their perceptions of cancer care needs for Indigenous people more broadly rather than specific to each dominant Indigenous group in the province; that is, First Nations and Métis. Therefore, participants’ discussion was predominantly focused on the barriers for First Nations patients and consequently, the recommendations to improving cancer care exclude the unique barriers that Métis experience in the health care system. Rather than asking questions from a pan-Indigenous perspective, future research should emphasize asking questions that distinguish between Indigenous groups because of the practical differences in experiences and access to cancer care. Due to our time constraints arising from conducting interviews with service providers during their workday, we were limited by our capacity to probe for how issues (e.g., travel, Elder support) may differ for First Nations and Métis cancer patients.

Although the current study focused on the support needs of Indigenous cancer patients, the results revealed that service providers also experienced systemic barriers to delivering medical care to First Nations patients (e.g., advocating for necessary supports for sometimes extensive wait times through the NIHB program). This finding is not surprising considering the reality of a fragmented and multi-jurisdictional health care system for registered First Nations. Future research could include an assessment of barriers specific to service providers through interviews with providers, policy makers, and health care administrators, or a program evaluation of Indigenous-specific medical coverage organizations to determine and address systemic issues that impede health care professionals’ ability to provide care to Indigenous patients.

## Conclusions

An advisory team of Indigenous patient partners, clinicians, academics, and policy makers identified that a research priority was to conduct a needs assessment of Indigenous cancer care in Saskatchewan from the perspective of cancer service providers. The results of the needs assessment identified gaps in supports and outlined recommendations to address barriers to cancer care for Indigenous patients and their families affected by cancer. These recommendations may inform evidence-based health system interventions for Indigenous cancer patients and ultimately aim to improve cancer care services, quality of life, and health outcomes of Indigenous patients throughout their cancer journey.

## Supplementary Information

**Additional file 1.**

## Data Availability

Data supporting the findings are outlined in the manuscript. For the privacy of the participants, transcripts of the data are not publicly available.

## Abbreviations

COPS: Chemotherapy Outreach Program of Saskatchewan
NIHB: Non-Insured Health Benefits
SCA: Saskatchewan Cancer Agency
SHA: Saskatchewan Health Authority

## Footnotes

1 The language of Cree people, a First Nations people in Canada. In Saskatchewan, they inhabit territories from the northern boreal forest to the southern plains.

2 Dene are a First Nations people who inhabit territory in the boreal forest (such as northern Saskatchewan) and Arctic regions of Canada.

## References

[1] Elias B, Kliewer EK, Hall M, Demers AA, Turner D, Martens P, et al. The burden of cancer risk in Canada’s indigenous population: a comparative study of known risks in a Canadian region. Int J Gen Med. 2011;4:699–709. 10.2147/ijgm.s24292. 10.2147/IJGM.S24292 PMC3206113 22069372

[2] Métis Nation of Ontario and Cancer Care Ontario. Cancer in the Métis people of Ontario: risk factors and screening behaviours. 2015. http://www.metisnation.org/media/653628/mno-cco-report-screen.pdf. Accessed 07 Dec 2020.

[3] Withrow DR, Pole JD, Nishri ED, Tjepkema M, Marrett LD (2017). Cancer survival disparities between first nation and non-Aboriginal adults in Canada: follow-up of the 1991 census mortality cohort. Cancer Epidemiol Prevention Biomarkers.

[4] Marret L, Jones CR, Wishart K. First Nations cancer research and surveillance priorities for Canada. Workshop Report. 2004. Cancer Care Ontario.

[5] Jamal S, Jones C, Walker J, Mazereeuw M, Sheppard AJ, Henry D, et al. [Internet]. Cancer in First Nations people in Ontario, Canada: Incidence and mortality, 1991 to 2010. Government of Canada, Statistics Canada; 2021 [cited 2021Jul15]. Available from: https://www150.statcan.gc.ca/n1/pub/82-003-x/2021006/article/00002-eng.htm 10.25318/82-003-x202100600002-eng 34142787

[6] Horrill TC, Dahl L, Sanderson E, Munro G, Garson C, Taylor C, Fransoo R, Thompson G, Cook C, Linton J, Schultz AS. Comparing cancer incidence, stage at diagnosis and outcomes of first nations and all other Manitobans: a retrospective analysis. BMC Cancer 2019;19(1):1–0. 10.1186/s12885-019-6296-7 PMC6836370 31694679

[7] Horrill TC, Dahl L, Sanderson E, Munro G, Garson C, Fransoo R, Thompson G, Cook C, Linton J, Schultz AS (2019). Cancer incidence, stage at diagnosis and outcomes among Manitoba first nations people living on and off reserve: a retrospective population-based analysis. CMAJ Open.

[8] Canada. Non-insured health benefits for First Nations and Inuit. 2020. https://www.sac-isc.gc.ca/eng/1572537161086/1572537234517. Accessed 08 Dec 2020.

[9] Horrill TC, Linton J, Lavoie JG, Martin D, Wiens A, Schultz AS (2019). Access to cancer care among indigenous peoples in Canada: a scoping review. Soc Sci Med.

[10] Statistics Canada. Data from Statistics Canada: focus on geography series, 2016 census. Statistics Canada Catalogue no. 98–404-X2016001. Ottawa, Ontario. 2017.

[11] Métis Nation of Alberta. Medical accommodations. 2020. http://albertametis.com/medical-accommodations/. Accessed 30 June 2020.

[12] Carr T, Sedgewick JR, Roberts R, Arcand L, Ali A, Groot G. A patient-oriented study of Indigenous peoples’ experiences of cancer in Saskatchewan: a qualitative narrative analysis using sharing circles. CMAJ Open. 2020;8(4):E852. 10.9778/cmajo.20200012 PMC7881746 33303571

[13] Groot G, Waldron T, Barreno L, Cochran D, Carr T. Trust and world view in shared decision making with indigenous patients: a realist synthesis. J Eval Clin Pract. 2020;26(2):503–14. 10.1111/jep.13307 PMC7154772 31750600

[14] Letendre A, Garvey G, King A, King M, Crowshoe R, Bill L (2020). Creating a Canadian indigenous research network against cancer to address indigenous cancer disparities. JCO Global Oncol.

[15] Carr T, Sedgewick JR, Roberts R, Groot G. The sharing circle method: understanding Indigenous cancer stories. In SAGE Research Methods Cases. 2020. https://dx-doi-org.ezproxy.library.yorku.ca/10.4135/9781529711264.

[16] Stevens A, Raftery J, Mant J, Simpson S. Health care needs assessment: the epidemiologically based needs assessment reviews. Vol. 1. Radcliffe Publishing; 2004.

[17] Saskatchewan Health Authority. First Nations and Métis health service - about us. 2015. https://www.saskatoonhealthregion.ca/locations_services/Services/fnmh/service. Accessed 15 Sept 2020.

[18] Morgan DL (1996). Focus groups as qualitative research.

[19] Davidson PM, Halcomb EJ, Gholizadeh L (2013). Focus groups in health research.: foundations for evidence-based practice.

[20] Faulkner SL, Trotter SP. Theoretical saturation. The International Encyclopedia of Communication Research Methods 2017:1–2.

[21] Groot G, Needs assessment by service providers for indigenous Cancer supports in Saskatchewan. Research Report 2020 June. https://static1.squarespace.com/static/5b22e5239d5abb6a167c048c/t/5f74b5b811eb1c0f757bdcd1/1601484219014/Needs+Assessment+by+Service+Providers+for+Indigenous+Cancer+Supports+in+Saskatchewan.pdf;

[22] Braun V, Clarke V (2006). Using thematic analysis in psychology. Qual Res Psychol.

[23] Groot G (2020). An environmental scan of provincial cancer supports for indigenous patients and their families in Saskatchewan.

[24] Maar M, Burchell A, Little J, Ogilvie G, Severini A, Yang JM, Zehbe I (2013). A qualitative study of provider perspectives of structural barriers to cervical cancer screening among first nations women. Womens Health Issues.

[25] Johnston G, Vukic A, Parker S (2013). Cultural understanding in the provision of supportive and palliative care: perspectives in relation to an indigenous population. Brit Med J Support Palliative Care.

[26] Hunter G, Sanchez M. Child and family poverty in Saskatchewan: 2019 Research report. Socl Policy Res Centre 2019. https://www.uregina.ca/socialwork/sprc/assets/2019-Saskatchewan-Child-Poverty-Report.pdf

[27] McCallum MJL (2017). Starvation, experimentation, segregation, and trauma: words for reading indigenous health history. Can Historical Review.

[28] Moffatt J, Mayan M, Long R (2013). Sanitoriums and the Canadian colonial legacy: the untold experiences of tuberculosis treatment. Qual Health Res.

[29] Tang SY, Browne AJ (2008). ‘Race’ matters: racialization and egalitarian discourses involving Aboriginal people in the Canadian health care context. Ethn Health.

[30] McConkey S (2017). Indigenous access barriers to health care services in London, Ontario. Univ West Ont Med J.

[31] Roberts RA. Stories about cancer among the woodland Cree of northern Saskatchewan (doctoral dissertation). University of Saskatchewan 2005.

[32] Roberts RA, Groot G, Carr T (2020). Decisions on cancer care by indigenous peoples in Alberta and Saskatchewan: a narrative analysis. Rural Remote Health.

