# Supplementary material for: Service providers’ perceptions of support needs for Indigenous cancer patients in Saskatchewan: a needs assessment
Source: BMC Health Serv Res. 2021 Aug 21;21:848. doi: 10.1186/s12913-021-06821-6 (PMC8380380; doi:10.1186/s12913-021-06821-6)
Supplement: Supplementary file 1 — Additional file 1. [file 12913_2021_6821_MOESM1_ESM.docx]

**Appendix: Group Interview Questions**

The following questions do not have a right or wrong answer, so please free to express your opinions openly. My role is to facilitate a smooth functioning of this group interview. Please use your pseudonym and position in your workplace when you would like to answer a question.

1. How do you know when your patient is Indigenous?

2. What formal supports exist in your workplace for Indigenous patients?

- At the time of diagnosis?
- During treatment?
- What aftercare/follow-up services are available?
- How effective or helpful are local formal supports?

3. What informal supports (e.g., Elders, homecare workers, lay-counsellors) exist in your workplace for Indigenous patients?

- At the time of diagnosis?
- During treatment?
- What aftercare/follow-up services are available?
- How effective or helpful are local informal supports?

4. From your point of view as (ask for person’s position), what do you think your workplace needs regarding formal supports for Indigenous patients with cancer and their families?

- Around the time of diagnosis?
- During treatment?
- What aftercare/follow-up formal supports are needed in your community?

5. From your point of view as (ask for person’s position), what do you think your workplace needs regarding informal supports for Indigenous patients with cancer and their families?

- Around the time of diagnosis?
- During treatment?
- What aftercare/follow-up informal supports are needed in communities?

6. From the formal support needs you have identified, which ones are the most important to improve Indigenous patient care and family experiences?

7. From the informal support needs you have identified, which ones are the most important to improve Indigenous patient care and family experiences?

8. When it comes to formal supports, what barriers or obstacles affect their usefulness?

9. When it comes to informal supports, what barriers or obstacles affect their usefulness?

10. Do you have anything else you’ve like to add about the cancer care needs of Indigenous patients in your workplace?
